# Electrified interfaces from first-principles: continuum electrochemistry and grand canonical density-functional theory

**DOI:** 10.1038/s41524-026-02200-z

**Published:** 2026-07-11

**Authors:** Mouyi Weng, Nicephore Bonnet, Oliviero Andreussi, Nicola Marzari

**Affiliations:** 1 Theory and Simulations of Materials (THEOS), and National Centre for Computational Design and Discovery of Novel Materials (MARVEL), Ecole Polytechnique Federale de Lausanne, 1015 Lausanne, Switzerland; 2 Department of Chemistry and Biochemistry, Boise State University, Boise, ID USA; 3 Laboratory for Materials Simulations (LMS), Paul Scherrer Institut (PSI), CH-5232 Villigen, PSI Switzerland

**Keywords:** Chemistry, Materials science, Physics

## Abstract

Simulating electrochemical interfaces within density-functional theory (DFT) requires incorporating the electrochemical potential. In practice, this is commonly achieved by varying the number of electrons in the Kohn–Sham equations, leading to grand-canonical DFT calculations. The grand-canonical formalism is related to the canonical ensemble through a Legendre transformation, which maps the energy onto the grand potential while leaving the corresponding atomic forces unchanged. Building on these established energy and force relations, we investigate how higher-order derivatives, including vibrational response properties, are modified under grand-canonical conditions. We derive an explicit correction term that relates the grand-canonical and canonical force-constant matrices. The correction is validated using finite-difference calculations for CO adsorbed on Pt(111), where it explains the ensemble dependence of the vibrational Stark tuning rate, *S*_*ω*_ = *dω*/*dU*. The derived expression also explains why vibrational modes involving motion perpendicular to the surface exhibit larger ensemble-dependent shifts than modes dominated by parallel motion. It also rationalizes the thermodynamic-limit behavior, in which ensemble differences vanish as the adsorbate coverage decreases. Finally, we discuss the limitations of grand-canonical calculations combined with implicit-solvent models, showing that Stark tuning rates depend sensitively on solvent and electrolyte-cavity parameters.

## Introduction

Simulating electrochemical interfaces using density-functional theory (DFT) requires a description of the electrode potential^1–4^. For reaction thermodynamics, the computational hydrogen electrode (CHE) remains the most widely used approximation^5,6^. CHE treats the electron and proton reservoirs through an electron-reservoir thermodynamic cycle, so that an elementary free-energy change contains a term proportional to the number of transferred electrons and the electrode potential^7^. This construction is most reliable when the interfacial electronic structure is not strongly altered by the transferred charge, for example, in the dilute-site or thermodynamic-limit regime. Within this reservoir picture, electrochemical reaction energies are naturally described through the Legendre transform between the canonical energy and the grand-canonical potential.

For systems in which charge transfer substantially changes the interfacial electronic structure, grand-canonical DFT provides a direct way to treat the electrode as an electron reservoir at controlled electrochemical potential^8–11^. It has become an increasingly used approach for studying electrode charging, interfacial energetics, and electrocatalytic reactions^12–21^. Grand-canonical simulations can be obtained either by Legendre-transforming a series of canonical calculations at prescribed electron numbers^22^, or by imposing the target Fermi level directly in the self-consistent cycle while allowing the electron number to adjust^23^. The latter strategy has motivated direct grand-canonical implementations in several electronic-structure codes, including JDFTx^23^, CP2K^24^, GPAW^25^, and the effective screening medium (ESM) method in Quantum ESPRESSO^26^. Our group has contributed to first-principles approaches for electrified interfaces^27,28^, to implicit-solvent and continuum-electrolyte descriptions of the electric double layer^22,29–35^, and to grand-canonical energetics obtained by Legendre-transforming canonical calculations^30^. More recently, starting from Quantum ESPRESSO 7.3^34,36^ with Quantum ENVIRON 3.1^30^, the self-consistent grand-canonical implementation was made compatible with SCCS solvation and continuum-electrolyte screening; the calculations below use this combined scheme.

The thermodynamic basis of these calculations is the Legendre transform. For a fixed atomic configuration, it maps the canonical energy at the corresponding electron number to the grand-canonical potential at the imposed Fermi level. Differentiating this transformed potential shows that the first derivative of the grand potential with respect to atomic coordinates is equal to the canonical force evaluated at the corresponding charge^37,38^. This property underlies the use of canonical calculations to locate equilibrium structures, transition states, free-energy differences, and activation barriers on grand-canonical potential-energy surfaces. However, this first-derivative equivalence does not automatically extend to response functions involving higher derivatives.

Vibrational frequencies are second derivatives of the energy with respect to atomic displacements. In this work, we examine how canonical and grand-canonical ensembles affect these response properties. We derive analytical relationships between canonical and grand-canonical force constants and show that their difference is governed by charge-displacement responses and by the interfacial chemical hardness, or inverse differential capacitance. We first validate the correction for CO adsorbed on Pt(111) by reconstructing grand-canonical vibrational frequencies from canonical finite-difference data and comparing them with explicit grand-canonical calculations. We also examine ensemble effects in potential-dependent vibrational spectroscopy, summarized by the Stark tuning rate,

1 $${S}_{\omega }=\frac{d\omega }{dU},$$

where *ω* is a vibrational frequency and *U* is the electrode potential. We then use two examples to interpret the origin of the ensemble difference: the different responses of CO vibrational modes perpendicular and parallel to the Pt surface, and the finite-cell trend toward the thermodynamic limit. In that limit, the vibrating-adsorbate coverage ratio approaches zero, the displacement-induced Fermi-level shift vanishes, and the canonical and grand-canonical force constants converge. Because the one-CO-per-cell size series lowers the CO coverage at the same time, we also add fixed-coverage 4 × 4 control calculations with four CO molecules to compare a local single-CO displacement with a collective all-CO displacement, thereby changing the vibrating-CO coverage ratio while holding the total CO coverage fixed. Finally, we compare calculated Stark tuning rates with experimental results and examine their sensitivity to continuum-solvent and electrolyte-cavity parameters, emphasizing which conclusions are formal consequences of the ensemble transformation and which are model-dependent quantitative comparisons.

## Results

### Grand-canonical energies, forces and vibrational properties

We compare response properties obtained from canonical and grand-canonical DFT. The two formalisms use different thermodynamic control variables: the electron number in the canonical ensemble and the electrochemical potential in the grand-canonical ensemble. These variables are thermodynamically conjugate, so the canonical energy and the grand-canonical potential are connected by a Legendre transform at corresponding state points. This connection makes the first derivatives with respect to atomic coordinates identical, but it does not make the higher-order response functions identical because the controlled electronic variable also responds to the atomic displacement. Following previous work on grand-canonical forces^37,38^, we first recapitulate the Legendre transform and force relation in the notation used here, so that the subsequent force-constant derivation is self-contained.

We first discuss the total energy of the system, starting from its expression in the canonical ensemble, *E*^*C*^(*α*, *N*_*e*_), as a function of the atomic degrees of freedom, *α*, and the number of electrons, *N*_*e*_. For a re-optimized metallic or fractional-occupation energy curve, the derivative with respect to total electron number defines the electronic chemical potential, which we write as the Fermi energy,

2 $${\left(\frac{\partial {E}^{C}(\alpha ,{N}_{e})}{\partial {N}_{e}}\right)}_{\alpha }={E}_{f}\equiv {E}_{f}^{* }(\alpha ,{N}_{e})$$

This relation is consistent with Janak’s theorem in the sense that the change in the re-optimized total energy with fractional occupation is governed by frontier Kohn–Sham eigenvalues, but Eq.(2) is used here as the thermodynamic definition of the canonical electronic chemical potential. The superscript * indicates that, when computed from simulations in the canonical ensemble, the Fermi energy becomes a function of the canonical natural variables. By a Legendre transform, we define the grand-canonical potential as

3 $${E}^{GC}(\alpha ,{E}_{f})={E}^{C}(\alpha ,{N}_{e})-{N}_{e}{E}_{f},$$

where now the Fermi energy is the natural state variable. In a self-consistent grand-canonical calculation, the electron number is the value ${N}_{e}^{* }(\alpha ,{E}_{f})$ that makes the canonical electronic chemical potential equal to the imposed Fermi level. Thus, if we fix the Fermi energy in a grand-canonical calculation and use the corresponding number of electrons in a canonical simulation, the resulting Fermi energy recovers the imposed value, i.e.,

4 $${E}_{f}^{* }(\alpha ,{N}_{e}^{* }(\alpha ,{E}_{f}))={E}_{f}$$

and, vice versa,

5 $${N}_{e}^{* }(\alpha ,{E}_{f}^{* }(\alpha ,{N}_{e}))={N}_{e}.$$

The above identities express the invertibility of the Legendre transform between the conjugate variables *N*_*e*_ and *E*_*f*_.

Because either conjugate variable can be used to parameterize the same state, Eq.(3) can be ambiguous in practical applications. It is therefore useful to write the grand-canonical potential explicitly in terms of its natural variable, namely

6 $${E}^{GC}(\alpha ,{E}_{f})={E}^{C}(\alpha ,{N}_{e}^{* }(\alpha ,{E}_{f}))-{N}_{e}^{* }(\alpha ,{E}_{f}){E}_{f}.$$

Taking the partial derivative of Eq.(6) with respect to one of the atomic coordinates allows us to derive the relationship between interatomic forces in the two ensembles. Leveraging the fact that the atomic displacements are not explicit functions of the conjugate variables involved in the Legendre transform, we have

7 $${\left(\frac{\partial {E}^{GC}(\alpha ,{E}_{f})}{\partial {\alpha }_{I\mu }}\right)}_{{E}_{f}}={\left(\frac{\partial {E}^{C}(\alpha ,{N}_{e})}{\partial {\alpha }_{I\mu }}\right)}_{{N}_{e} = {N}_{e}^{* }(\alpha ,{E}_{f})}$$

which implies that the forces in the grand-canonical ensemble can be expressed directly in terms of the ones from the canonical ensemble. The subscript on the canonical derivative specifies that the derivative is evaluated at the electron number corresponding to the grand-canonical state at the imposed Fermi level *E*_*f*_,

8 $${f}_{I\mu }^{GC}(\alpha ,{E}_{f})={f}_{I\mu }^{C}(\alpha ,{N}_{e}^{* }(\alpha ,{E}_{f})).$$

Following the above derivation, we can investigate the relationship between the second derivatives of the energy with respect to atomic displacements in the two ensembles. The matrix of force constants in the grand-canonical ensemble is given by

9 $${c}_{IJ\mu \nu }^{GC}\left(\alpha ,{E}_{f}\right)={\left(\frac{\partial }{\partial {\alpha }_{J\nu }}\frac{\partial }{\partial {\alpha }_{I\mu }}{E}^{GC}\left(\alpha ,{E}_{f}\right)\right)}_{{E}_{f}}={\left(-\frac{\partial }{\partial {\alpha }_{J\nu }}{f}_{I\mu }^{GC}\left(\alpha ,{E}_{f}\right)\right)}_{{E}_{f}},$$

where *I* and *J* are atom indices, while *μ* and *ν* indicate the Cartesian directions (x, y, and z). Exploiting the result in Eq. (8) we can show that

10 $${c}_{IJ\mu \nu }^{GC}\left(\alpha ,{E}_{f}\right)={\left.{\left(\frac{{\partial }^{2}{E}^{C}\left(\alpha ,{N}_{e}\right)}{\partial {\alpha }_{J\nu }\partial {\alpha }_{I\mu }}\right)}_{{N}_{e}}\right\vert }_{{N}_{e} = {N}_{e}^{* }(\alpha ,{E}_{f})}+{\left(\frac{\partial {N}_{e}^{* }\left(\alpha ,{E}_{f}\right)}{\partial {\alpha }_{J\nu }}\right)}_{{E}_{f}}{\left.{\left(\frac{\partial {E}_{f}^{* }\left(\alpha ,{N}_{e}\right)}{\partial {\alpha }_{I\mu }}\right)}_{{N}_{e}}\right\vert }_{{N}_{e} = {N}_{e}^{* }(\alpha ,{E}_{f})}$$

The first term on the right-hand side of Eq. (10) corresponds to the canonical force constants, while the second term involves the derivative of the Legendre product between conjugate variables. By exploiting Eqs.(4) and (5), we can rewrite Eq. (10) in a more symmetric form as

11 $$\begin{array}{rcl}{c}_{IJ\mu \nu }^{GC}\left(\alpha ,{E}_{f}\right)&=&{c}_{IJ\mu \nu }^{C}\left(\alpha ,{N}_{e}^{* }\left(\alpha ,{E}_{f}\right)\right)-\\ &&{\left(\frac{\partial {N}_{e}^{* }\left(\alpha ,{E}_{f}\right)}{\partial {E}_{f}}\right)}_{\alpha }\cdot {\left(\frac{\partial {E}_{f}^{* }\left(\alpha ,{N}_{e}\right)}{\partial {\alpha }_{J\nu }}\right)}_{{N}_{e}}\cdot {\left(\frac{\partial {E}_{f}^{* }\left(\alpha ,{N}_{e}\right)}{\partial {\alpha }_{I\mu }}\right)}_{{N}_{e}}\end{array}$$

12 $$\begin{array}{rcl}{c}_{IJ\mu \nu }^{GC}\left(\alpha ,{E}_{f}\right)&=&{c}_{IJ\mu \nu }^{C}\left(\alpha ,{N}_{e}^{* }\left(\alpha ,{E}_{f}\right)\right)-\\ &&{\left(\frac{\partial {E}_{f}^{* }\left(\alpha ,{N}_{e}\right)}{\partial {N}_{e}}\right)}_{\alpha }\cdot {\left(\frac{\partial {N}_{e}^{* }\left(\alpha ,{E}_{f}\right)}{\partial {\alpha }_{J\nu }}\right)}_{{E}_{f}}\cdot {\left(\frac{\partial {N}_{e}^{* }\left(\alpha ,{E}_{f}\right)}{\partial {\alpha }_{I\mu }}\right)}_{{E}_{f}}\end{array}$$

In Eqs.(11) and (12), all response functions are evaluated at the matched canonical and grand-canonical state defined by ${N}_{e}={N}_{e}^{* }(\alpha ,{E}_{f})$ and the imposed *E*_*f*_.

Equations (10), (11), and (12) reveal that the grand-canonical force-constant matrix, and consequently the vibrational frequencies, can differ from the canonical force-constant matrix. The origin of this difference is the variation of the electron number as atoms move in the grand-canonical ensemble, or equivalently, the variation of the Fermi level as atoms move in the canonical ensemble. Because the differential capacitance is positive for the stable interfaces considered here, Eq.(12) shows that the correction is negative semidefinite in the subspace spanned by the charge-displacement response ${(\partial {N}_{e}^{* }/\partial {\alpha }_{I\mu })}_{{E}_{f}}$. Modes that induce little charge redistribution are therefore nearly ensemble independent, while modes that strongly modulate the interfacial dipole are softened in the grand-canonical ensemble. An equivalent relation has also been reported in ref. ^39^. Moreover, the equations provide a practical bridge between the two thermodynamic frameworks: force constants in one ensemble can be reconstructed from calculations performed in the other when the capacitance and charge-response derivatives are known.

From a practical standpoint, directly evaluating the correction term remains challenging. While continuum embedding methods have been combined with linear response density-functional perturbation theory for electronic excitations ^40^, no comparable implementation exists for atomic displacements. Therefore, in the following, we employ a finite-difference approach to compute force-constant matrices in both canonical and grand-canonical ensembles.

### Surface capacitance and chemical hardness

Eqs.(12) and (11) involve the changes in the chemical potential or number of electrons as one atom of the system is moved, while keeping the conjugate variable fixed. The factor multiplying these terms can be associated with either the differential capacitance

13 $$C\left({E}_{f}\right)={\left(\frac{\partial {N}_{e}^{* }\left(\alpha ,{E}_{f}\right)}{\partial {E}_{f}}\right)}_{\alpha }$$

or the chemical hardness of the electrified interface

14 $$\eta \left(\alpha ,{E}_{f}\right)={\left.{\left(\frac{\partial {E}_{f}^{* }\left(\alpha ,{N}_{e}\right)}{\partial {N}_{e}}\right)}_{\alpha }\right\vert }_{{N}_{e} = {N}_{e}^{* }(\alpha ,{E}_{f})}=\frac{1}{C\left({E}_{f}\right)}.$$

For a fixed geometry, we suppress the explicit *α* dependence below. Leveraging the derivation of Binninger^41^, we can express the chemical hardness of an electrified interface in the presence of a continuum description of the electrolyte (dielectric screening and ionic countercharge) as the sum of three terms

15 $$\eta \left({E}_{f}\right)={\eta }_{Q}\left({E}_{f}\right)+{\eta }_{XC}\left({E}_{f}\right)+{\eta }_{DL}\left({E}_{f}\right).$$

The quantum hardness is related to the finite-temperature density of states of the electronic system, ${g}_{D}^{T}\left({E}_{f}\right)$, as

$${\eta }_{Q}\left({E}_{f}\right)=\frac{1}{{g}_{D}^{T}\left({E}_{f}\right)},$$

The exchange-correlation hardness can be expressed as

16 $${\eta }_{XC}=\int\frac{{g}_{LD}^{T}\left({E}_{f},{\bf{r}}\right)}{{g}_{D}^{T}\left({E}_{f}\right)}\left(\int\frac{\delta {\mu }_{XC}\left({\bf{r}}\right)}{\delta {\rho }^{elec}\left({\bf{r}}^{\prime} \right)}{f}^{elec}\left({\bf{r}}^{\prime} \right)d{\bf{r}}^{\prime} \right)d{\bf{r}},$$

where ${\mu }_{XC}\left({\bf{r}}\right)$ is the exchange-correlation functional used in the electronic structure calculation, ${g}_{LD}^{T}\left({E}_{f},{\bf{r}}\right)$ is the local density of states at the Fermi energy, and

17 $${f}^{elec}\left({\bf{r}}\right)=\frac{\partial {\rho }^{elec}\left({\bf{r}}\right)}{\partial N},$$

is the electronic Fukui function of the system.

Eventually, the electrostatic component of the chemical hardness, coming from the electrostatic potential due to the electrons and the continuum diffuse layer charge reorganization, is expressed as

18 $${\eta }_{DL}=\int\frac{{g}_{LD}^{T}\left({E}_{f},{\bf{r}}\right)}{{g}_{D}^{T}\left({E}_{f}\right)}\left({\phi }^{f}\left({\bf{r}}\right)-{\phi }_{b}^{f}\right)d{\bf{r}},$$

with

$${\phi }^{f}=\int\frac{{f}^{elec}\left({\bf{r}}{\prime} \right)-{f}^{DL}\left({\bf{r}}{\prime} \right)}{\left\vert {\bf{r}}-{\bf{r}}{\prime} \right\vert }d{\bf{r}}{\prime}$$

being the electrostatic potential generated by the electronic and diffuse-layer Fukui functions, with an asymptotic value of ${\phi }_{b}^{f}$ in the bulk of the electrolyte. Similar to the electronic Fukui function, the diffuse-layer counterpart is defined as

19 $${f}^{DL}\left({\bf{r}}\right)=\frac{\partial {\rho }^{diel}\left({\bf{r}}\right)}{\partial N}+\frac{\partial {\rho }^{ion}\left({\bf{r}}\right)}{\partial N}.$$

It accounts for the charge flow in the continuum environment as the charge of the substrate is changed, as imposed by the requirement of charge neutrality in the simulation cell, with

20 $$\int\,{f}^{elec}\left({\bf{r}}\right)d{\bf{r}}=1\quad \,\mathrm{and}\,\quad \int\,{f}^{DL}\left({\bf{r}}\right)d{\bf{r}}=1.$$

When the metal is sufficiently thick, the density of states at the Fermi energy is large, and the quantum hardness becomes small. Moreover, because the electronic Fukui function is localized near the metal electrode surface and the normalized local density of states (LDOS) is periodic within the bulk of the electrode, the exchange-correlation contribution also becomes small for thick metallic slabs. The Fermi-level LDOS maps in Fig. S6 show that the states available for infinitesimal charging are mainly distributed inside the Pt slab for the 2 × 2, 3 × 3, and 4 × 4 cells, consistent with treating the five-layer slab as sufficiently metallic for the present scaling analysis. In this limit the diffuse-layer term dominates and approaches the potential drop, Δ*ϕ*^*f*^, induced by the electrochemical Fukui densities:

21 $${\eta }_{DL}=\frac{1}{{C}_{DL}}=\Delta {\phi }^{f}\propto \frac{1}{A}\int\,z\left({f}^{elec}\left({\bf{r}}{\prime} \right)-{f}^{DL}\left({\bf{r}}{\prime} \right)\right)d{\bf{r}}$$

where *A* is the surface area of the slab. This term is proportional to the average surface dipole generated by these densities. Therefore, for a sufficiently thick metal slab and a fixed local interfacial perturbation, the chemical hardness is inversely proportional to the surface area, while the surface capacitance is directly proportional to the surface area.

### Finite-difference validation for CO on Pt(111)

We use carbon monoxide adsorbed on Pt(111) as a model system because CO vibrational spectroscopy is a sensitive probe of the electrostatic environment at metal-electrolyte interfaces^42^. We computed finite-difference vibrational frequencies for one CO molecule on 2 × 2, 3 × 3, and 4 × 4 Pt(111) surface cells. Since each supercell contains one CO molecule, increasing the lateral cell size simultaneously decreases the coverage from 1/4 to 1/9 and 1/16 monolayer. The calculations, therefore, test whether the predicted trend appears in realistic finite cells, but they do not by themselves disentangle finite-size effects from coverage-dependent adsorbate-adsorbate interactions, adsorbate dipole density, changes in the potential of zero charge, or changes in interfacial capacitance.

We first validate Eq.(11) by comparing grand-canonical vibrational frequencies reconstructed from canonical finite-difference data with frequencies obtained from explicit grand-canonical finite-difference calculations. This comparison tests whether the correction term, rather than a numerical artifact of the finite-difference procedure, accounts for the observed ensemble dependence.

**Fig. 1:**
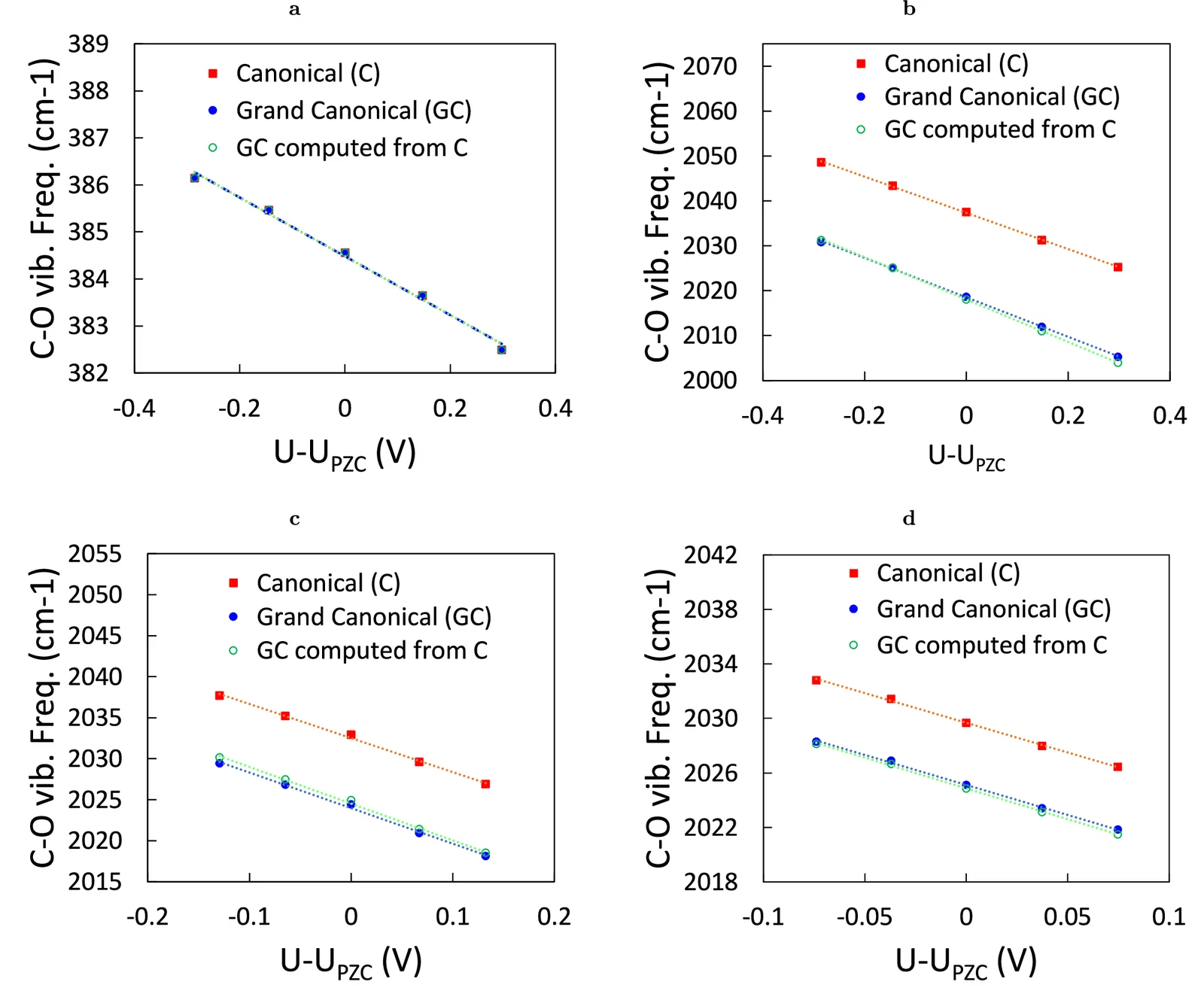
Vibrational frequencies of selected modes as a function of the Fermi energy. **a** Modes in the xy plane (parallel) for 1/4 CO coverage; **b** modes in the z-direction (perpendicular) for 1/4 CO coverage; **c** z-direction modes for 1/9 CO coverage; **d** z-direction modes for 1/16 CO coverage. Frequencies computed directly under grand-canonical and canonical finite-difference conditions are shown in blue and red, respectively. Grand-canonical frequencies reconstructed from canonical data using Eq.(11) are shown in green. Linear fits are used to extract Stark tuning rates from all three data sources; rates are reported with the electrochemical-potential sign convention defined in Eq.(1).

Figure 1 summarizes the calculated vibrational frequencies. The red and blue markers are the explicit canonical and grand-canonical finite-difference results, while the green markers are the grand-canonical frequencies reconstructed from canonical data using the force-constant correction.

The reconstructed frequencies track the explicit grand-canonical finite-difference frequencies shown in blue across the sampled potential range. This agreement validates the correction term and supports the interpretation that the observed frequency shifts are the ensemble correction described by Eqs.(11) and (12).

With this validation in hand, we interpret the remaining trends through the charge-displacement response. Eqs.(11) and (12) show that the ensemble dependence of vibrational frequencies is controlled by how strongly an atomic displacement changes the Fermi level in the canonical ensemble or the electron number in the grand-canonical ensemble. Motions parallel to the surface only weakly change the adsorbate-metal separation and should have small ${(\partial {N}_{e}^{* }/\partial \alpha )}_{{E}_{f}}$, whereas motions perpendicular to the surface change the interfacial dipole and should couple more strongly to the electrode charge. Because the correction in Eq.(12) is negative for a positive hardness, the grand-canonical force constant should be smaller than the canonical one for modes with appreciable charge response.

For the 2 × 2 cell, the parallel modes in Fig. 1a show almost no ensemble dependence: the canonical and grand-canonical frequencies differ by only about 0.1 cm^−1^ over the sampled potentials, and the extracted Stark slopes are nearly identical. This is the expected behavior for modes with negligible charge-displacement response.

The perpendicular modes show a qualitatively different behavior. In the 2 × 2 cell, the canonical frequency is on average 18.8 cm^−1^ higher than the grand-canonical frequency (Fig. 3a). This sign is consistent with Eq.(12): the grand-canonical correction softens modes that induce charge redistribution. The ensemble also changes the Stark tuning rate of the perpendicular mode, with the canonical and grand-canonical slopes differing by 3.7cm^−1^V^−1^ in the default continuum model (Fig. 3b).

The mode-projected data in Table S1 quantify this contrast. For the 2 × 2 cell, the parallel modes have *D*_*q*_ < 7.69 × 10^−5^*e*/Å, close to the finite-difference numerical precision, whereas the perpendicular mode has *D*_*q*_ = 1.117*e*/Å. The corresponding predicted ensemble frequency shifts are below 10^−6^ cm^−1^ for the parallel modes and 18.36 cm^−1^ for the perpendicular mode.

The size series probes how the correction changes when the vibrating-CO coverage ratio decreases. For a localized displacement, the change in Fermi energy with respect to atomic displacement, ${\left(\partial {E}_{f}^{* }(\alpha ,{N}_{e})/\partial {\alpha }_{I\mu }\right)}_{{N}_{e}}$, is expected to scale as 1/*A*. By contrast, the number of electrons exchanged with the reservoir in the grand-canonical ensemble is proportional to the capacitance times the induced potential shift, and therefore remains approximately finite for a localized adsorbate perturbation. Eqs.(11) and (12) then imply that the difference between the canonical and grand-canonical force constants should scale approximately as 1/*A* in a fixed-coverage thermodynamic-limit analysis. Thus, for a local vibrational perturbation on a macroscopic electrode, the displacement-induced Fermi-level shift vanishes, and the canonical and grand-canonical vibrational responses converge.

**Fig. 2:**
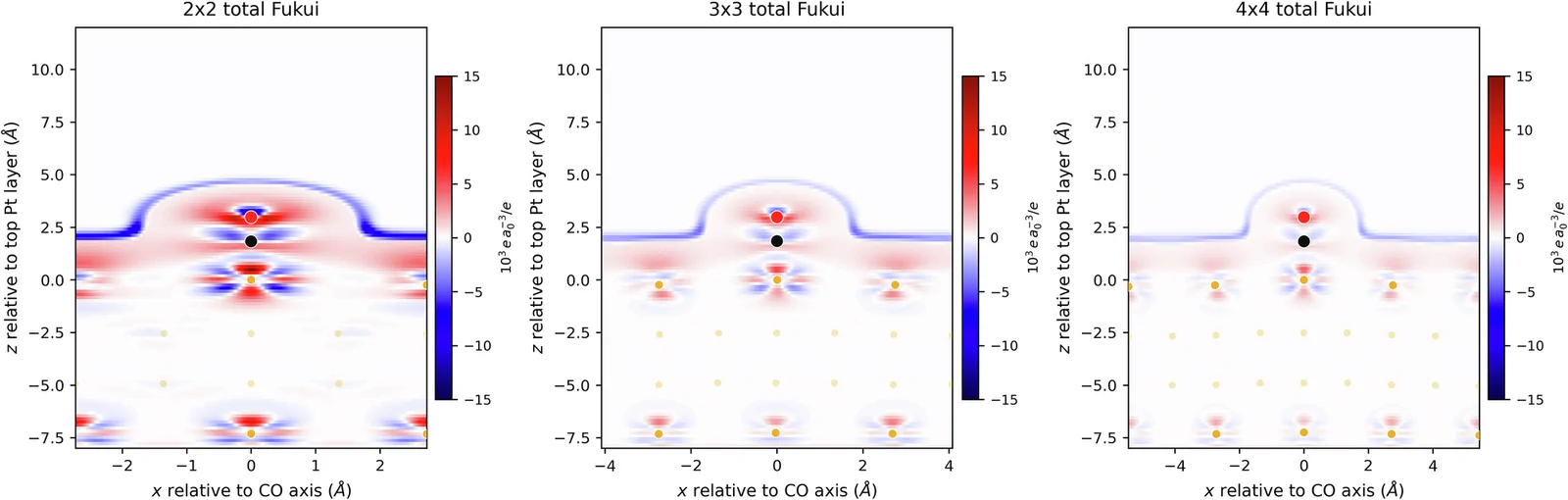
Total electrochemical Fukui response for CO/Pt(111) in the 2 × 2, 3 × 3, and 4 × 4 cells. The plotted quantity is *f*^*tot*^(**r**) = *f*^*elec*^(**r**) + *f*^*DL*^(**r**), shown in an *x* − *z* plane through the CO axis with *z* = 0 at the top Pt layer. Black dots mark C atom positions and red dots mark O atom positions. All panels use the same color scale, so the decreasing intensity from 2 × 2 to 4 × 4 directly visualizes the reduction of the local charge response associated with the vibrating-CO coverage ratio.

The average frequency difference decreases for larger supercells, from 18.8 cm^−1^ in the 2 × 2 cell to 7.36 and 4.55 cm^−1^ in the 3 × 3 and 4 × 4 cells, respectively. This trend is consistent with a decreasing vibrating-CO coverage ratio and with the expected approach toward ensemble equivalence in the thermodynamic limit. Figure 2 provides the corresponding spatial picture: using a shared color scale, the total electrochemical Fukui response around the CO/Pt interface becomes progressively weaker from the 2 × 2 to the 4 × 4 cell. At the same time, the decreasing total CO coverage changes lateral CO–CO interactions, adsorbate dipole density, and possibly the potential of zero charge and differential capacitance.

However, because the one-CO-per-cell size series increases the surface area and lowers the total CO coverage simultaneously, the interpretation of the decreasing ensemble difference requires an additional check. In particular, one may ask whether the smaller canonical–grand-canonical frequency difference in larger cells is mainly caused by weaker lateral CO–CO interactions at lower coverage, or by the larger surface area, equivalently, the smaller vibrating-CO coverage ratio, entering the force-constant correction. To address this question, we performed additional fixed-coverage controls in a 4 × 4 cell containing four CO molecules, corresponding to the same 1/4 monolayer total CO coverage as the original 2 × 2 cell. The data are reported in Table S2. We considered two z-direction vibrational subspaces. In the first, all four CO molecules were allowed to vibrate along the surface normal, giving a vibrating-CO coverage ratio of 1/4. This collective control is the fixed-coverage analog of the original 2 × 2 periodic calculation for the in-phase CO-stretch branch. In the second, only the C and O atoms of one CO molecule were displaced along z, while the other three CO molecules remained fixed, giving a vibrating-CO coverage ratio of 1/16. This local control keeps the total CO coverage fixed but reduces the coverage ratio of the vibrating perturbation.

The fixed-coverage controls support the trend expected from the force-constant correction. In the local single-CO control, the ensemble shift is close to the original one-CO 4 × 4 value and much smaller than the original 2 × 2 value. By contrast, in the all-CO control, the highest CO-stretch branch gives an ensemble shift close to the original 2 × 2 result. These controls indicate that the vibrating-CO coverage ratio is the dominant factor for the ensemble difference, while lateral CO-CO coupling is secondary for the local single-CO stretch.

The charge-response quantities in Table S1 support the same interpretation. As the CO coverage decreases from 1/4 to 1/9 and 1/16 in the one-CO-per-cell size series, the cell-level chemical hardness decreases from 2.915 to 1.310 and 0.743 eV/*e*^2^, respectively. This decrease reflects the larger surface area and associated increase in capacitance. By contrast, the local perpendicular charge response remains nearly constant, *D*_*q*_ ≈ 1.12 *e*/Å, while the parallel response is below 8 × 10^−5^ *e*/Å for the 2 × 2 cell. Combining these values through $\Delta {k}_{q}=-\eta {D}_{q}^{2}$ predicts decreasing perpendicular frequency shifts of 18.36, 8.40, and 4.73 cm^−1^. Therefore, the reduction of the canonical–grand-canonical vibrational-frequency difference with decreasing coverage is mainly caused by the larger surface area and the corresponding lower chemical hardness, rather than by a strong change in the local CO–CO interaction.

**Fig. 3:**
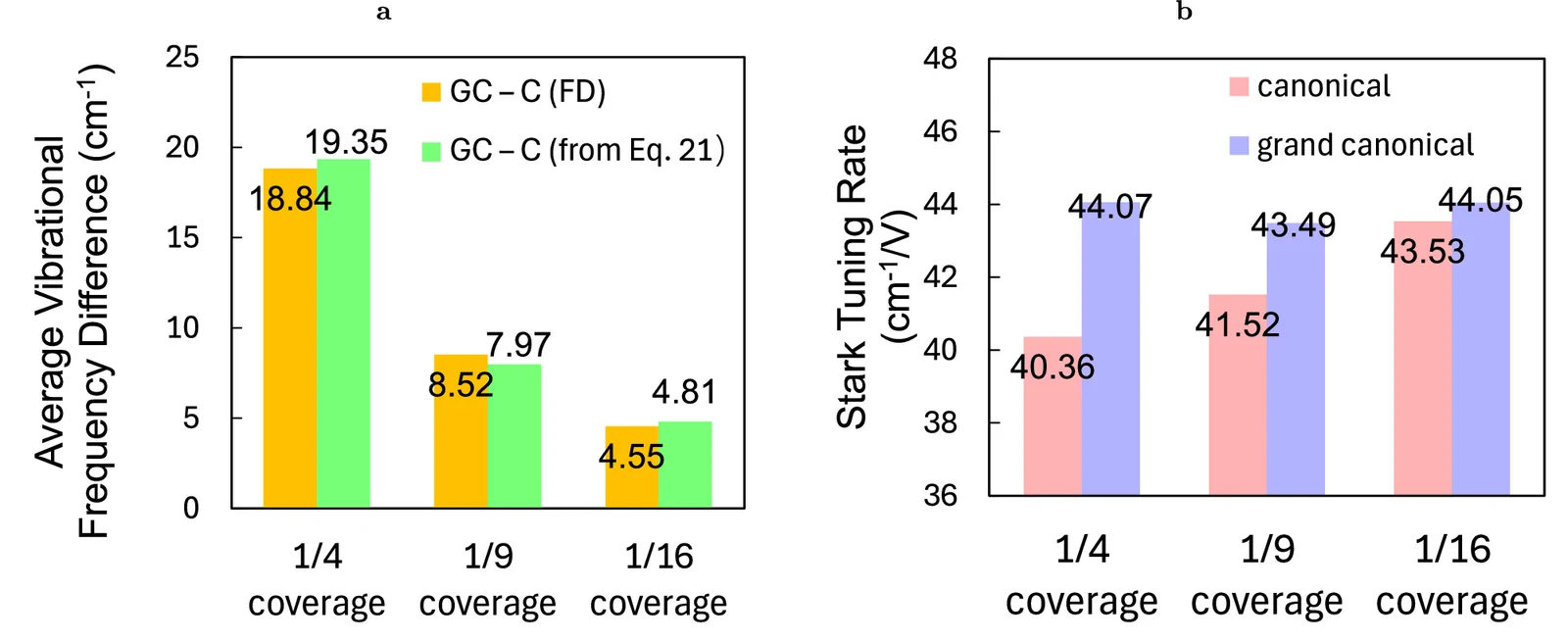
Comparison of vibrational properties computed under canonical and grand-canonical ensembles for Pt(111) surfaces of different coverage (1/4, 1/9, and 1/16). **a** Average vibrational frequency difference between the two ensembles. Orange bars represent values directly calculated via finite-difference (FD) methods, while green bars are obtained by applying Eq.(11) to canonical results to compute grand-canonical frequencies. **b** Stark tuning rates, extracted from the slopes of vibrational frequencies as a function of the Fermi energy, under both canonical (red bars) and grand-canonical (blue bars) conditions.

The same qualitative trend appears in the Stark tuning rates shown in Fig. 3b. The ensemble dependence is small for modes parallel to the surface, but appreciable for perpendicular modes and reduced in the larger, lower-coverage cells. Table 1 summarizes the key quantities used to discuss this trend, including the potential of zero charge (PZC) and differential capacitance extracted for each cell.

**Table 1:** Summary of quantities extracted for the CO/Pt(111) size series

Cell	CO coverage	〈Δ*ω*〉	${S}_{\omega }^{GC}$	${S}_{\omega }^{C}$	PZC	*C*_diff_
		(cm^−1^)	(cm^−1^V^−1^)	(cm^−1^V^−1^)	(V)	(*μ*F cm^−2^)
2 × 2	1/4	18.8	44.07	40.37	5.816	10.71
3 × 3	1/9	7.36	43.49	41.52	5.983	10.59
4 × 4	1/16	4.55	44.05	43.53	6.017	10.50

〈Δ*ω*〉 denotes the average canonical-minus-grand-canonical frequency difference for the perpendicular modes. ${S}_{\omega }^{GC}$ and ${S}_{\omega }^{C}$ are Stark tuning rates obtained from linear fits using the sign convention of Eq.(1). PZC and *C*_diff_ were evaluated for the same interfacial model and coverage as the vibrational calculations.

### Interpreting Stark tuning rates at electrochemical interfaces

The preceding section establishes the ensemble correction to vibrational force constants. We now discuss Stark tuning rates, which are useful for comparison with electrochemical vibrational spectroscopy but are more model dependent. Experimental Stark tuning rates for CO on Pt are typically around 29 cm^−1^ V^−1^ ^43–45^. With the default continuum parameters used here, the calculated perpendicular-mode slopes are 44.07 cm^−1^ V^−1^ in the grand-canonical ensemble and 40.37 cm^−1^ V^−1^ in the canonical ensemble for the 2 × 2 cell. The ensemble difference is a direct consequence of the force-constant correction discussed above, whereas the absolute agreement with experiment depends on the electrochemical interface model.

One source of model dependence is the dielectric response of interfacial water. Molecular simulations and experiments indicate that the effective dielectric response normal to the surface-confined water layer can be much smaller than the bulk value of 78, with representative values in the range 4–9^46,47^. A continuum model with a spatially uniform bulk permittivity, therefore, cannot simultaneously represent both the interfacial water layer and the bulk electrolyte. This limitation affects quantitative Stark slopes because the vibrational frequency is sensitive to the local electric field at the adsorbate.

As a sensitivity test, we repeated the calculation with *ϵ*_0_ = 6, within the reported interfacial range. The resulting Stark tuning rates, shown in Fig. 5a, are 28.4cm^−1^V^−1^ in the grand-canonical ensemble and 24.4cm^−1^V^−1^ in the canonical ensemble. These values are closer to the experimental range, but they should be interpreted as evidence of parameter sensitivity rather than as a calibrated prediction. A spatially varying dielectric model or an explicit interfacial water layer would be needed for a more controlled quantitative comparison.

**Fig. 4:**
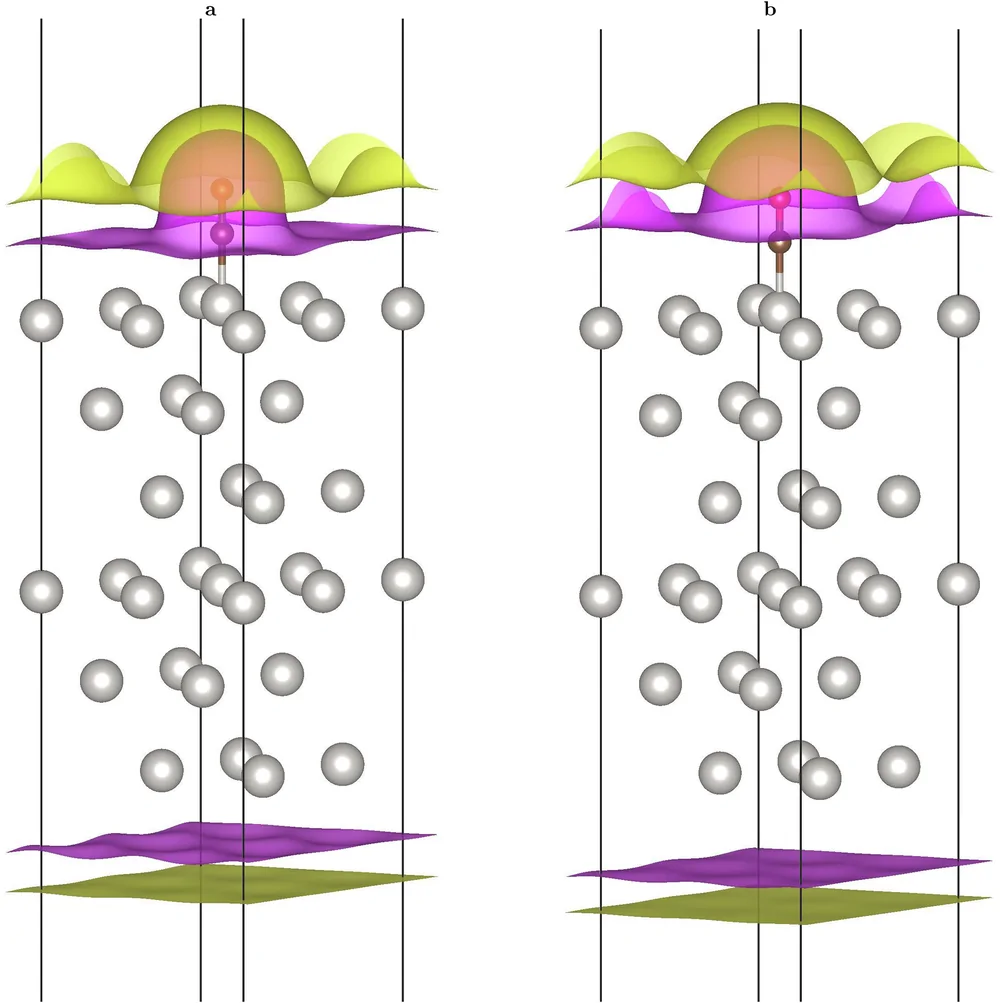
Continuum interfaces of the selected test systems. Atomic structure of the selected test systems (round spheres) together with the continuum interfaces adopted for the solvent, in panel (**a**), and the electrolyte, in panel (**b**). For the solvent’s continuum interface, the reported yellow and purple iso-surfaces correspond to *ρ*_*min*,*ϵ*_ = 1.2 × 10^−4^ a.u. and *ρ*_*max*,*ϵ*_ = 2.2 × 10^−3^ a.u., lying approximately at distances of 2.0 and 3.1 Å from the Pt surface, respectively. For the electrolyte’s continuum interface, the yellow and violet iso-surfaces correspond to *ρ*_min,*γ*_ = 3 × 10^−5^ a.u. and *ρ*_max,*γ*_ = 4 × 10^−4^ a.u., lying approximately at distances of 2.6 and 3.8 Å from the Pt surface, respectively.

Another source of model dependence is the position of the solvent and electrolyte cavities. Implicit models replace microscopic solvent and ion configurations with mean-field dielectric and ionic charge distributions. Their interaction with the metal and adsorbate, therefore, depends on the switching parameters that determine where the continuum media begin. Previous work has calibrated related parameters against solvation energies, work functions in solution, surface energies, and differential capacitances^22,30,31^. In the default calculations, the solvent dielectric boundary is defined by *ρ*_*min*,*ϵ*_ = 1.2 × 10^−4^ a.u. and *ρ*_*max*,*ϵ*_ = 2.2 × 10^−3^ a.u., while the electrolyte boundary is defined by *ρ*_*min*,*γ*_ = 3 × 10^−5^ a.u. and *ρ*_*max*,*γ*_ = 4 × 10^−4^ a.u., as visualized in Fig. 4. These values were chosen so that the solvent and Poisson–Boltzmann continuum interfaces lie at distances from the Pt surface comparable to those used in earlier first-principles electrochemistry models by Dabo and co-workers^27,48^. They were not fitted to reproduce the experimental Stark tuning rate. The default model is therefore used mainly to illustrate the ensemble difference between canonical and grand-canonical vibrational frequencies, while the following tests illustrate how the absolute Stark slope changes with continuum-interface parameters.

**Fig. 5:**
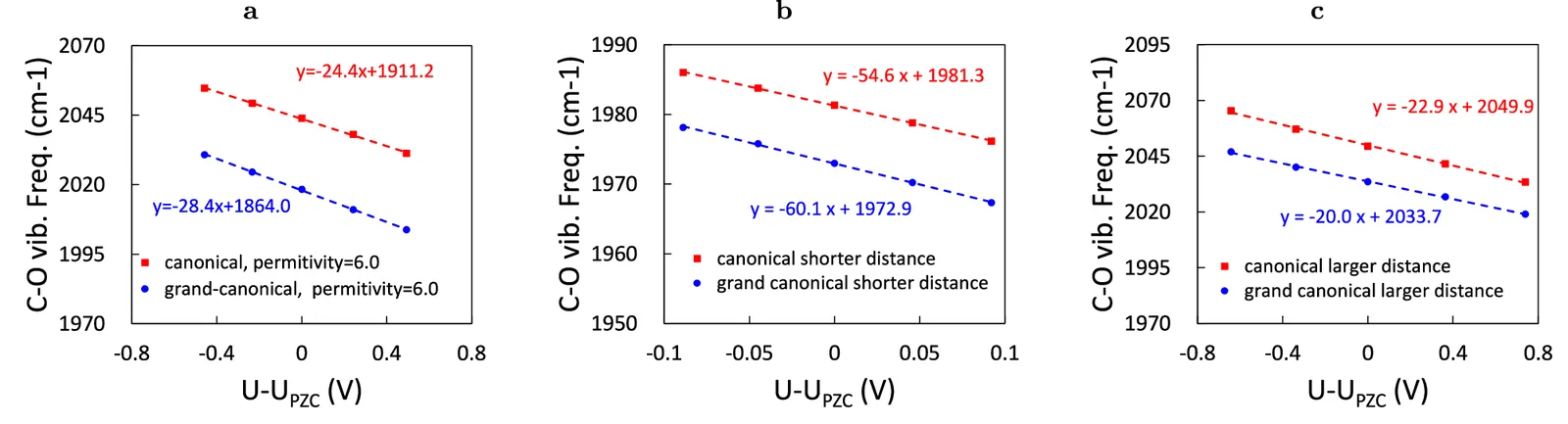
Sensitivity of calculated Stark tuning rates to continuum solvation and electrolyte parameters. **a** Stark tuning rates with *ϵ*_0_ = 6. **b** Stark tuning rates with shorter solvent and electrolyte interface distances. **c** Stark tuning rates with longer solvent and electrolyte interface distances.

In the first case, we used smaller interface distances: ${\rho }_{\min ,\epsilon }=1.2\times 1{0}^{-3}$ a.u. and ${\rho }_{\max ,\epsilon }=2.2\times 1{0}^{-2}$ a.u. for the solvent, and ${\rho }_{\min ,\gamma }=3\times 1{0}^{-4}$ a.u. and ${\rho }_{\max ,\gamma }=4\times 1{0}^{-3}$ a.u. for the electrolyte. These choices led to a higher surface capacitance of 34.5 μF/cm^2^, and the corresponding Stark tuning rates, shown in Fig. 5b, were 60.1 cm^−1^ V^−1^ in the grand-canonical ensemble and 54.6 cm^−1^ V^−1^ in the canonical ensemble.

In the second case, we used larger distances between the metal and both the implicit solvent and electrolyte regions. Specifically, for the solvent continuum interface, we set ${\rho }_{\min ,\epsilon }=3.0\times 1{0}^{-6}$ a.u. and ${\rho }_{\max ,\epsilon }=5.5\times 1{0}^{-5}$ a.u.; for the electrolyte continuum interface, we used ${\rho }_{\min ,\gamma }=5\times 1{0}^{-7}$ a.u. and ${\rho }_{\max ,\gamma }=5\times 1{0}^{-6}$ a.u. Under these settings, the computed surface capacitance was 4.5*μ*F/cm^2^. The resulting Stark tuning rates, shown in Fig. 5c, were 22.9 cm^−1^ V^−1^ in the grand-canonical ensemble and 20.0 cm^−1^ V^−1^ in the canonical ensemble.

These tests show that matching one observable does not guarantee quantitative transferability to another. A parameter set with a capacitance closer to the experimental range does not necessarily produce the best Stark tuning rate, and a parameter set with a lower capacitance can sometimes give a slope closer to experiment. The reason is that the capacitance probes the integral charge response of the interface, whereas the Stark tuning rate probes the local electric-field response at the adsorbate and its coupling to the C–O force constant.

These findings underscore that the absolute Stark tuning rates predicted by implicit electrochemical models are sensitive to dielectric constants, solvent-metal interface positions, and electrolyte profiles. The robust result of the present work is therefore not a particular fitted Stark slope, but the ensemble relation for force constants and the associated charge-response correction. Future models that combine explicit interfacial water with an implicit bulk electrolyte, or that allow spatially varying dielectric response, may be needed for predictive Stark slopes across different surfaces and coverages.

Finally, the grand-canonical and canonical ensembles can be viewed as two limiting descriptions of how the electrode charge responds during a vibration. The grand-canonical approach assumes that charge redistribution follows the nuclear displacement at constant Fermi level, whereas the canonical approach constrains the total electron number. The experimentally relevant limit depends on electronic relaxation and circuit response relative to the vibrational time scale. In the present calculations, the difference between these limits is largest for the high-coverage, small-cell models and becomes small at lower coverage, but a full dynamical treatment of charge transfer is beyond the scope of this work.

## Discussion

We have analyzed how canonical and grand-canonical ensembles affect vibrational response properties at electrified interfaces. The Legendre transform between canonical and grand-canonical descriptions maps the canonical energy to the grand-canonical potential at corresponding electron number and Fermi level, and the resulting first derivatives with respect to atomic coordinates are identical. This justifies the use of ordinary Hellmann–Feynman forces from canonical calculations for stationary structures on a grand-canonical surface. Taking one more derivative changes the situation: the force-constant matrix contains an additional term because either the electron number changes with atomic displacement in the grand-canonical ensemble or the Fermi level changes with atomic displacement in the canonical ensemble.

The finite-difference comparison provides the numerical validation of this correction: grand-canonical vibrational trends reconstructed from canonical data follow the explicit grand-canonical calculations. With this validation in hand, the formula gives a simple interpretation of the CO/Pt(111) results. Vibrations parallel to the surface barely change the surface dipole and are nearly ensemble independent, whereas perpendicular vibrations change the interfacial dipole and show a measurable frequency difference between the canonical and grand-canonical ensembles. For stable interfaces with positive capacitance, this behavior follows from the negative charge-response correction to modes that couple to charge redistribution.

The calculations also show a decreasing ensemble difference from the 2 × 2 to the 4 × 4 cell. This is consistent with reducing the vibrating-CO coverage ratio, which decreases the displacement-induced Fermi-level shift entering the correction. In the thermodynamic limit, where the vibrating-adsorbate coverage ratio approaches zero on a macroscopic electrode, this Fermi-level response should vanish and the canonical and grand-canonical force constants should coincide. The one-CO-per-cell series changes total CO coverage at the same time because each cell contains one CO molecule, but the fixed-coverage 4 × 4 controls help separate total-coverage effects from the vibrating-CO coverage-ratio effect. When only one CO molecule is allowed to vibrate along z in the fixed-coverage 4 × 4 cell, the ensemble shift is close to the original one-CO 4 × 4 value. When all four CO molecules are allowed to vibrate along z, the highest, in-phase-like stretch branch gives a shift close to the original 2 × 2 value. This comparison indicates that the vibrating-CO coverage ratio is more important for the ensemble difference than the lateral CO–CO interaction for the local stretch. A completely clean separation is still difficult in periodic supercell calculations because local and collective vibrational perturbations are not the same mode, but the control calculations support the coverage-ratio interpretation of the observed trend.

Stark tuning rates provide an experimentally relevant application of the same force-constant response, but their absolute values are sensitive to the continuum interface model. Changing the effective dielectric constant or the solvent/electrolyte cavity position shifts the calculated slopes substantially. These parameter sensitivities do not affect the formal ensemble relation for force constants, but they do limit quantitative comparisons between a static implicit-solvent model and experimental spectra. Real electrochemical surfaces may also contain defects, multiple adsorption geometries, dynamic solvent structures, and time-dependent charge relaxation that are absent from the present idealized Pt(111)/CO model.

More broadly, response properties in grand-canonical DFT require differentiating not only the explicit atomic dependence of the energy, but also the implicit dependence of electronic variables on the perturbation. Total energies and forces can be transferred between ensembles at corresponding state points, whereas second and higher derivatives require capacitance- and charge-response terms. This distinction is important for potential-dependent vibrational spectroscopy and should also be considered for other response properties at electrified interfaces.

## Methods

### Implicit solvent and electrolyte model

The electrostatic environment was described with the self-consistent continuum solvation model and a continuum electrolyte^30,31^. In vacuum, the electrostatic potential generated by the solute charge density satisfies

22 $${\nabla }^{2}{\Phi }^{tot}({\bf{r}})=-4\pi {\rho }^{solute}({\bf{r}})$$

where *Φ*^*tot*^(**r**) is the total electrostatic potential and *ρ*^*solute*^(**r**) is the charge density of the DFT subsystem. In the implicit-solvent calculation, the dielectric function *ϵ*(**r**) is spatially dependent, and the electrostatic potential is obtained from the generalized Poisson equation

23 $$\nabla \cdot \epsilon ({\bf{r}})\nabla {\Phi }^{tot}({\bf{r}})=-4\pi {\rho }^{solute}({\bf{r}}).$$

The solvent boundary is controlled by the density thresholds *ρ*_*max*,*ϵ*_ and *ρ*_*min*,*ϵ*_, which define the smooth transition from the DFT region to the bulk dielectric medium.

Electrolyte screening was included through a continuum ionic charge density. The generalized Poisson equation then becomes

24 $$\nabla \cdot \epsilon ({\bf{r}})\nabla \phi ({\bf{r}})=-4\pi [{\rho }^{solute}({\bf{r}})+{\rho }^{ion}({\bf{r}})]$$

with

25 $${\rho }^{ion}({\bf{r}})=\gamma ({\bf{r}})\mathop{\sum }\limits_{i}^{m}{Z}_{i}{c}_{i}[\phi ]({\bf{r}}),$$

where *Z*_*i*_ and *c*_*i*_(**r**) are the charge and concentration of ionic species *i*. The electrolyte interface function *γ*(**r**) confines the ionic charge to the continuum electrolyte region and is controlled by the switching parameters *ρ*_max,*γ*_ and *ρ*_min,*γ*_.

The ion concentrations are related to their bulk values, ${c}_{i}^{\infty }$, through the Boltzmann factor

26 $${c}_{i}[\phi ]({\bf{r}})={c}_{i}^{\infty }\cdot exp\left(-\frac{{Z}_{i}e\phi ({\bf{r}})}{kT}\right)$$

In this work, we used the linearized Poisson–Boltzmann form for numerical stability,

27 $${c}_{i}[\phi ]({\bf{r}})={c}_{i}^{\infty }\cdot \left(1-\frac{{Z}_{i}e\phi ({\bf{r}})}{kT}\right)$$

### Calculation details

All calculations were performed with Quantum ESPRESSO^34,36^ coupled to the Quantum ENVIRON library^30^. Norm-conserving SG15 pseudopotentials^49–51^ were used throughout. To ensure the accuracy of atomic forces and vibrational frequencies obtained from finite differences, we conducted convergence tests with respect to the plane-wave cutoff, vacuum thickness, metal slab thickness, and displacement step. The selected parameters reduce force errors to within 2 meV/Åand vibrational-frequency errors to below 2 cm^−1^. We also compared Hellmann–Feynman and finite-difference forces in the presence of the dielectric environment and the linearized Poisson–Boltzmann electrolyte; the difference is below 0.02 meV/Å. Details are provided in the Supporting Information.

The atomic structure, solvent boundary, and electrolyte boundary used in the simulations are visualized in Fig. 4. The default values were chosen by adjusting the solvent and Poisson–Boltzmann switching parameters so that the corresponding continuum surfaces are located at distances from the Pt surface comparable to those used in the first-principles electrochemistry model of Dabo and co-workers^27,48^. These parameters were not fitted to the Stark tuning rate, and they are used here primarily to demonstrate the canonical–grand-canonical vibrational-frequency difference within a representative implicit electrochemical model. In the simulations presented in the main size-series analysis, the solvent dielectric boundary is set by *ρ*_*min*,*ϵ*_ = 1.2 × 10^−4^ a.u. and *ρ*_*max*,*ϵ*_ = 2.2 × 10^−3^ a.u., while the electrolyte boundary is set by *ρ*_*min*,*γ*_ = 3 × 10^−5^ a.u. and *ρ*_*max*,*γ*_ = 4 × 10^−4^ a.u. The sensitivity to alternative cavity positions is reported in Fig. 5.

Vibrational frequencies were computed using a finite-difference approach implemented in the AiiDA ENVIRON workflow^52^ within the AiiDA framework^53,54^. To reduce the cost of the vibrational calculations, only the carbon and oxygen atoms were displaced when constructing the force-constant matrix. Owing to the large mass of Pt atoms, this approximation yields vibrational frequencies that agree with those obtained from a full dynamical matrix calculation involving the entire system^55^. To compare the two ensembles, canonical calculations were first performed at prescribed electron number, and grand-canonical calculations were carried out by imposing the Fermi energy obtained from the corresponding canonical calculation.

## Supplementary information

Supplementary Information

## Data Availability

The data that support the findings of this study are openly available via the Materials Cloud Archive (https://doi.org/10.24435/materialscloud:2s-gq). All simulations in this work were performed using open-source software. Density-functional theory calculations were carried out with the Quantum ESPRESSO package https://www.quantum-espresso.org. Implicit solvation calculations were performed using the ENVIRON module https://github.com/environ-developers/Environ. The AiiDA workflow developed in this work for the calculation of vibrational frequencies is publicly available at https://github.com/wengmouyi/aiida-environ-vib/releases/tag/paper-v1.

## Code availability

All simulations in this work were performed using open-source software. Density-functional theory calculations were carried out with the Quantum ESPRESSO package https://www.quantum-espresso.org. Implicit solvation calculations were performed using the ENVIRON module https://github.com/environ-developers/Environ. The AiiDA workflow developed in this work for the calculation of vibrational frequencies is publicly available at https://github.com/wengmouyi/aiida-environ-vib/releases/tag/paper-v1.
