# Supplementary material for: Electrified interfaces from first-principles: continuum electrochemistry and grand canonical density-functional theory
Source: NPJ Comput Mater. 2026 Jul 11;12(1):389. doi: 10.1038/s41524-026-02200-z (PMC13754575; doi:10.1038/s41524-026-02200-z)
Supplement: Supplementary file 1 [file 41524_2026_2200_MOESM1_ESM.pdf]

Supporting Information

Mouyi Weng,[1] Nicephore Bonnet,[1] Oliviero Andreussi,[2] and Nicola Marzari[1,3,*]

[1] *Theory and Simulations of Materials (THEOS), and National Centre for Computational Design and Discovery of Novel Materials (MARVEL), Ecole Polytechnique Federale de Lausanne, 1015 Lausanne, Switzerland*

[2] *Department of Chemistry and Biochemistry, Boise State University, Boise, Idaho 83725, USA*

[3] *Laboratory for Materials Simulations (LMS), Paul Scherrer Institut (PSI), CH-5232 Villigen PSI, Switzerland*

(Dated: May 31, 2026)

I. NUMERICAL CONVERGENCE TESTS

Finite-difference vibrational calculations are sensitive to numerical noise in the total energy and forces. We therefore tested the plane-wave cutoff, pseudopotential type, vacuum thickness, slab thickness, and finite-difference displacement step before evaluating the Stark tuning rates and canonical–grand-canonical frequency differences.

We first tested plane-wave cutoff energies and pseudopotentials in the DFT/SCCS framework. In reciprocal space, the dielectric functional $\varepsilon[\rho(\mathbf{r})]$ requires a higher cutoff than the electronic charge density $\rho(\mathbf{r})$, and both the polarization charge and total charge densities must be represented accurately. As shown in Fig. 1, vibrational frequencies systematically improve with increasing cutoff.

For these tests and subsequent calculations, we used SG15 norm-conserving pseudopotentials. Under the conventional relation $E_{\mathrm{cut}}^{\rho} = 4E_{\mathrm{cut}}^{\mathrm{wfc}}$, Fig. 1(a) shows that convergence is not reached even at wavefunction cutoffs as high as 150 Ry. We therefore fixed $E_{\mathrm{cut}}^{\rho} = 1200$ Ry and varied $E_{\mathrm{cut}}^{\mathrm{wfc}}$; Fig. 1(b) shows that convergence is reached for $E_{\mathrm{cut}}^{\mathrm{wfc}} > 80$ Ry. We then fixed $E_{\mathrm{cut}}^{\mathrm{wfc}} = 80$ Ry and varied E_{cut}^{ρ}, finding convergence for $E_{\mathrm{cut}}^{\rho} > 800$ Ry [Fig. 1(c)]. Ultrasoft pseudopotentials were also tested [Fig. 1(d)], but did not provide comparable convergence under the tested settings. We therefore used $E_{\mathrm{cut}}^{\mathrm{wfc}} = 80$ Ry and $E_{\mathrm{cut}}^{\rho} = 800$ Ry with norm-conserving pseudopotentials in the production calculations.

We next evaluated the effect of vacuum thickness, which is important for charged-slab calculations. Dabo's parabolic correction was applied to screen the charged metal surface[1]. As shown in Fig. 2, a vacuum thickness greater than 50 Å reduces atomic-force errors below 2 meV/Å and converges the vibrational frequency to within 0.2 cm^{-1}. We therefore used a 50 Å vacuum region in all production calculations.

We also tested the thickness of the Pt slab. Increasing the number of Pt layers from four to five shifts the absolute vibrational frequency by approximately 2 cm^{-1}, while changing the Stark tuning rate by less than 2 $\mathrm{cm}^{-1}\mathrm{V}^{-1}$, as shown in Fig. 3. We used five Pt layers in the main calculations.

For the finite-difference vibrational calculations, all atomic positions were first relaxed until each force was below 2 meV/Å. The carbon and oxygen atoms were then displaced along each Cartesian direction using a five-point finite-difference stencil. The displacement-

* nicola.marzari@epfl.ch

step test in Fig. 4 shows that the frequencies are robust over the range 3×10^{-5} Å to 3×10^{-3} Å, with errors below 1 cm^{-1}.

Finally, we validated the consistency between Hellmann–Feynman and finite-difference forces in the implicit solvation model, including dielectric response and the linearized Poisson–Boltzmann electrolyte. A single carbon atom on Pt(111) was displaced along the surface-normal direction. As shown in Fig. 5, the two force evaluations differ by less than 0.02 meV/Å.

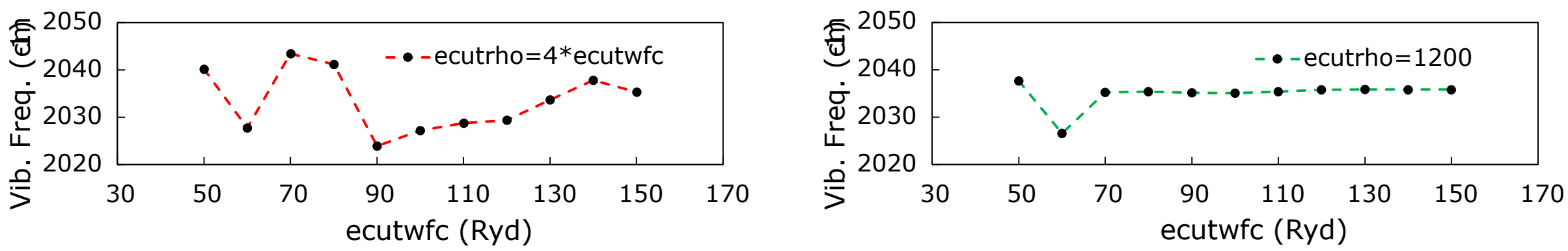

(a) Norm conserving, $E_{cut}^{\rho} = 4 \times E_{cut}^{wfc}$ (b) Norm conserving, $E_{cut}^{\rho} = 1200$ Ry

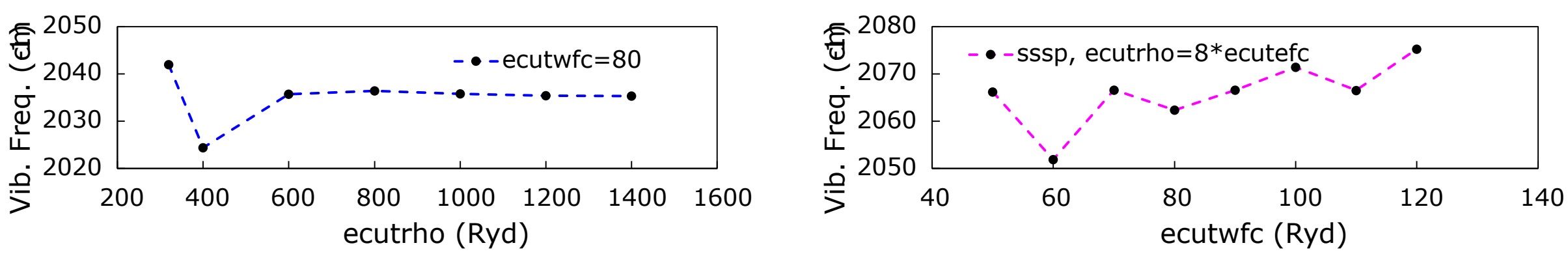

(c) Norm conserving, $E_{cut}^{wfc} = 80$ Ry (d) Ultrasoft, $E_{cut}^{\rho} = 8 \times E_{cut}^{wfc}$

FIG. 1. Convergence of the CO vibrational frequency with respect to plane-wave cutoff energies for the wavefunction and charge density.

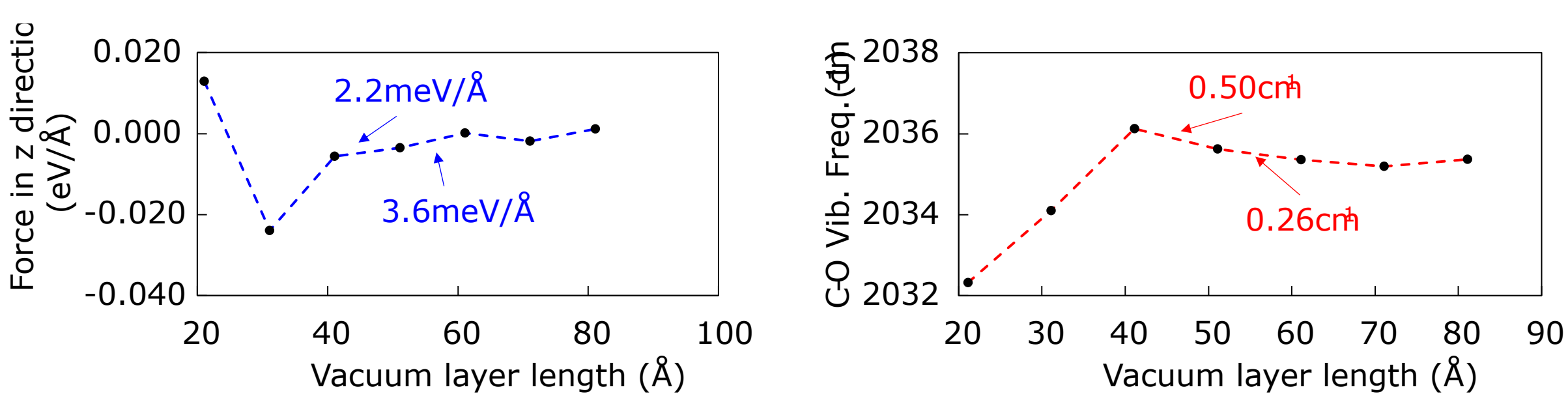

(a) Atomic-force convergence with vacuum thickness (b) Vibrational-frequency convergence with vacuum thickness

FIG. 2. Convergence with respect to vacuum thickness in the surface-normal direction. (a) Atomic force in the z direction. (b) CO vibrational frequency.

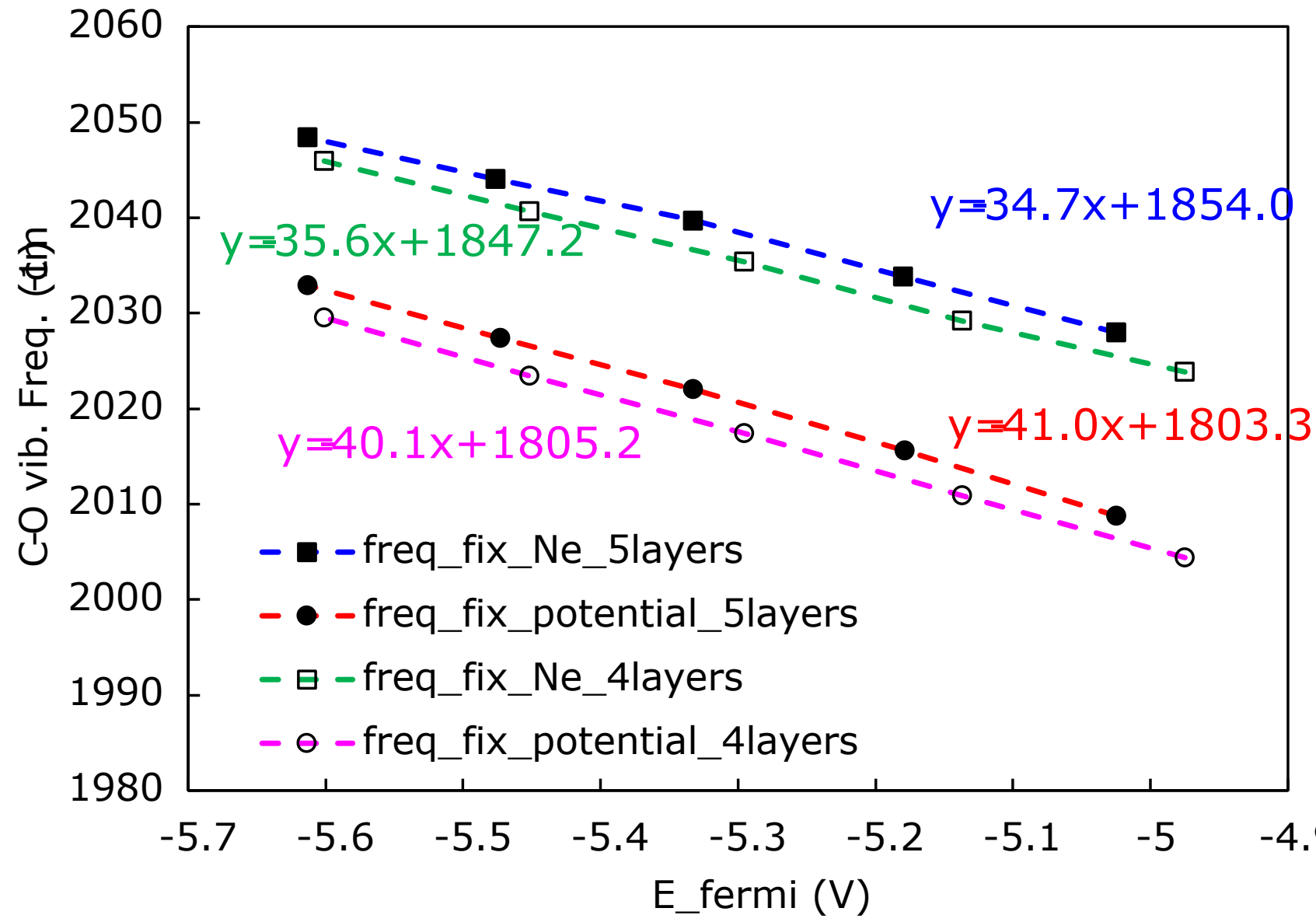

FIG. 3. Stark tuning rates of vibrational modes along the z direction. Green and violet dashed lines correspond to frequencies calculated with a four-layer Pt slab, while blue and red dashed lines correspond to a five-layer Pt slab.

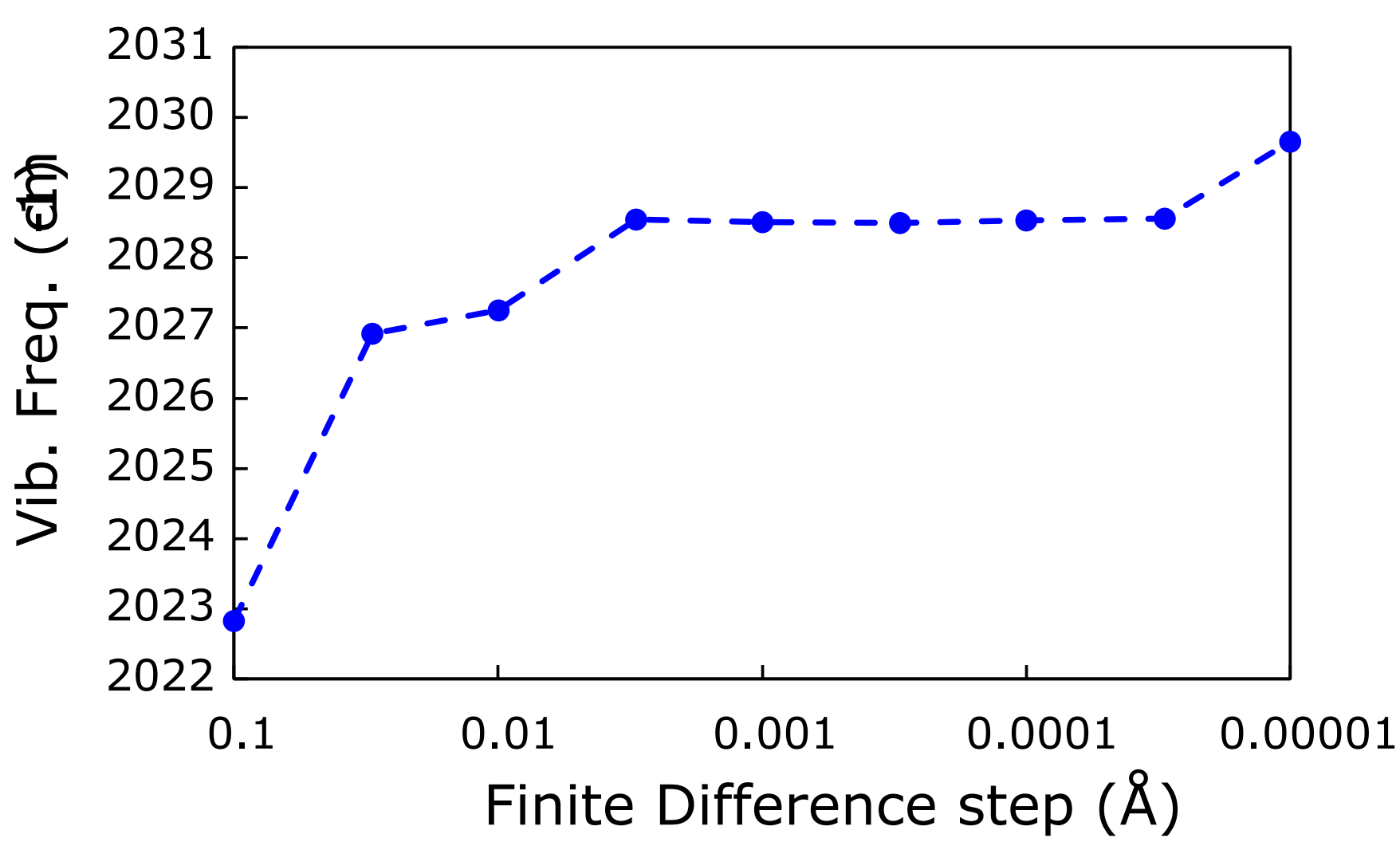

FIG. 4. Selected vibrational frequencies as a function of finite-difference displacement step.

II. CHARGE-RESPONSE ANALYSIS

The revised manuscript identifies the ensemble correction to the force-constant matrix as a product of the interfacial hardness and charge-displacement response. Table I projects

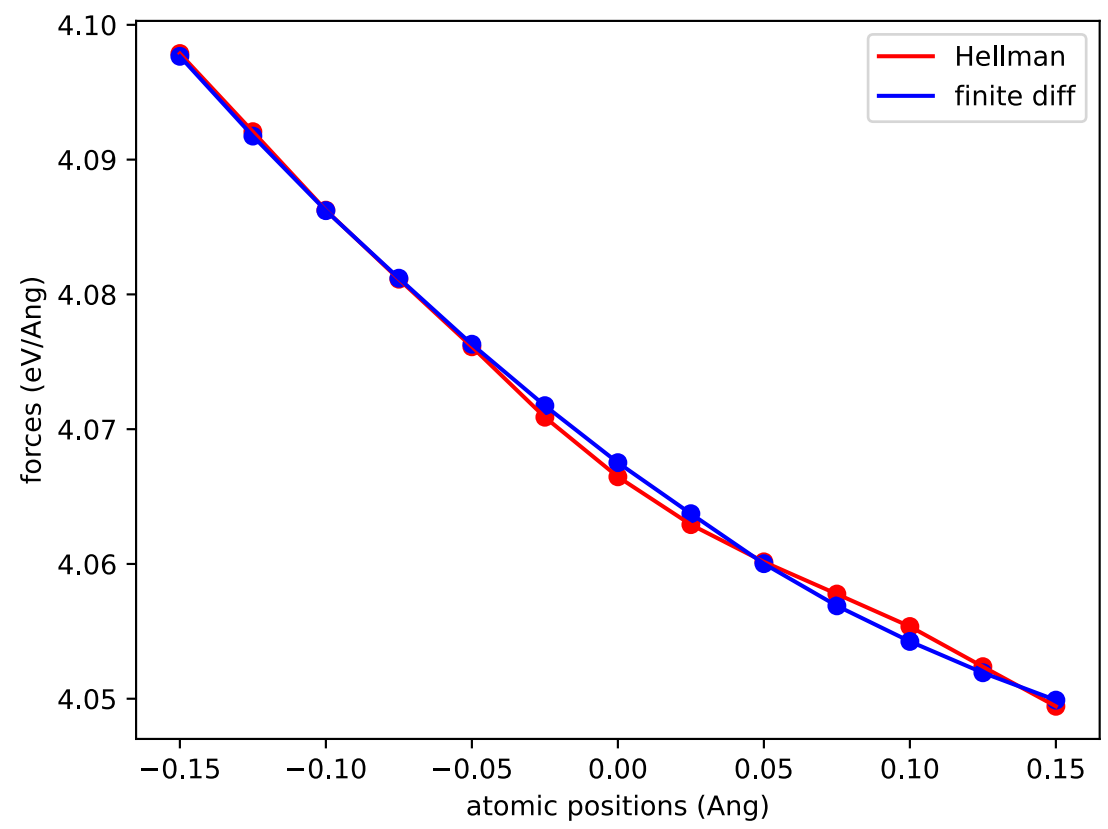

FIG. 5. Comparison between finite-difference and Hellmann–Feynman forces for a carbon atom on Pt(111), displaced along the z direction.

TABLE I. Quantities connecting the force-constant correction to the local electronic response. $D_q = (\partial N_e^*/\partial q)_{E_f}$ is the charge response along normal coordinate q, η is the cell-level interfacial chemical hardness, and $|\Delta k_q|$ is the magnitude of the projected correction to the mode force constant, with $\Delta k_q = -\eta D_q^2$. For the two parallel modes, the response is close to the finite-difference numerical precision, so upper bounds are reported.

Cell	Mode	D_q (e/Å)	η (eV/e^2)	$\|\Delta k_q\|$ (eV/Å^2)	$\Delta\omega_{\mathrm{pred}}$ (cm^{-1})	$\Delta\omega_{\mathrm{FD}}$ (cm^{-1})
2×2	parallel	$< 7.69 \times 10^{-5}$	2.915	$< 1.72 \times 10^{-8}$	$< 10^{-6}$	$< 5.1 \times 10^{-4}$
2×2	perpendicular	1.117	2.915	3.639	18.36	18.8
3×3	perpendicular	1.128	1.310	1.666	8.40	7.36
4×4	perpendicular	1.123	0.743	0.936	4.73	4.55

this correction onto the CO vibrational modes and compares the predicted frequency shifts with the explicit finite-difference results.

The projected charge response is negligible for the two parallel CO modes in the 2×2 cell, but is approximately 1.12 e/Å for the perpendicular mode across all cell sizes. The decreasing ensemble correction in the larger cells is therefore mainly caused by the decrease in the cell-level chemical hardness, rather than by a strong change in the local perpendicular charge response.

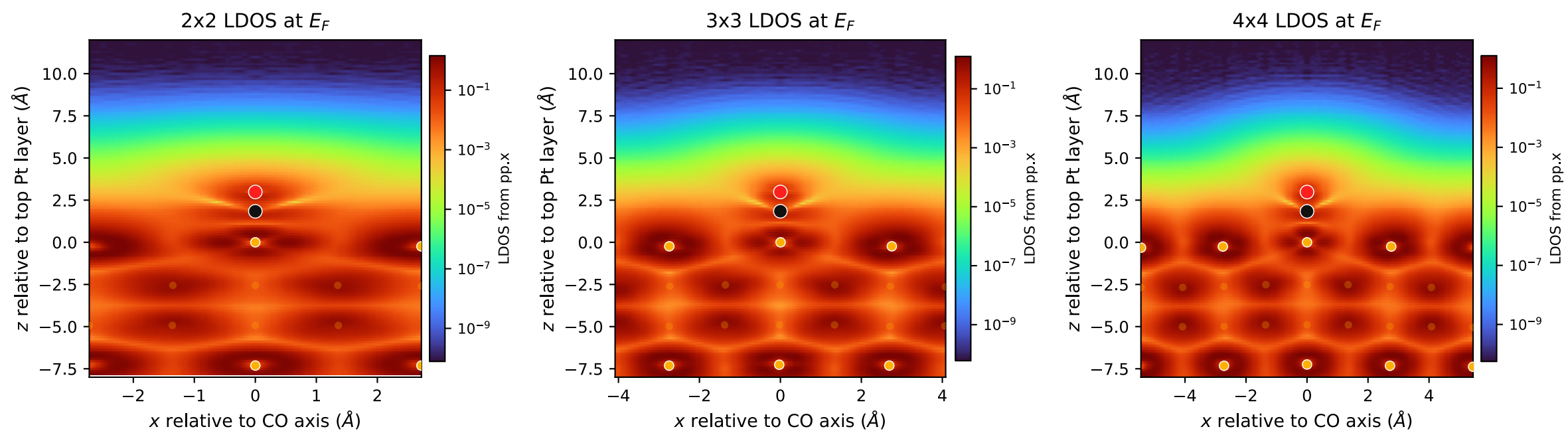

FIG. 6. Local density of states at the Fermi level, $g^T_{LD}(E_f, \mathbf{r})$, for CO/Pt(111) in the 2×2, 3×3, and 4×4 cells. The LDOS maps diagnose the metallic states available for infinitesimal charging. The plotted x-z plane passes through the CO axis, with $z = 0$ at the top Pt layer.

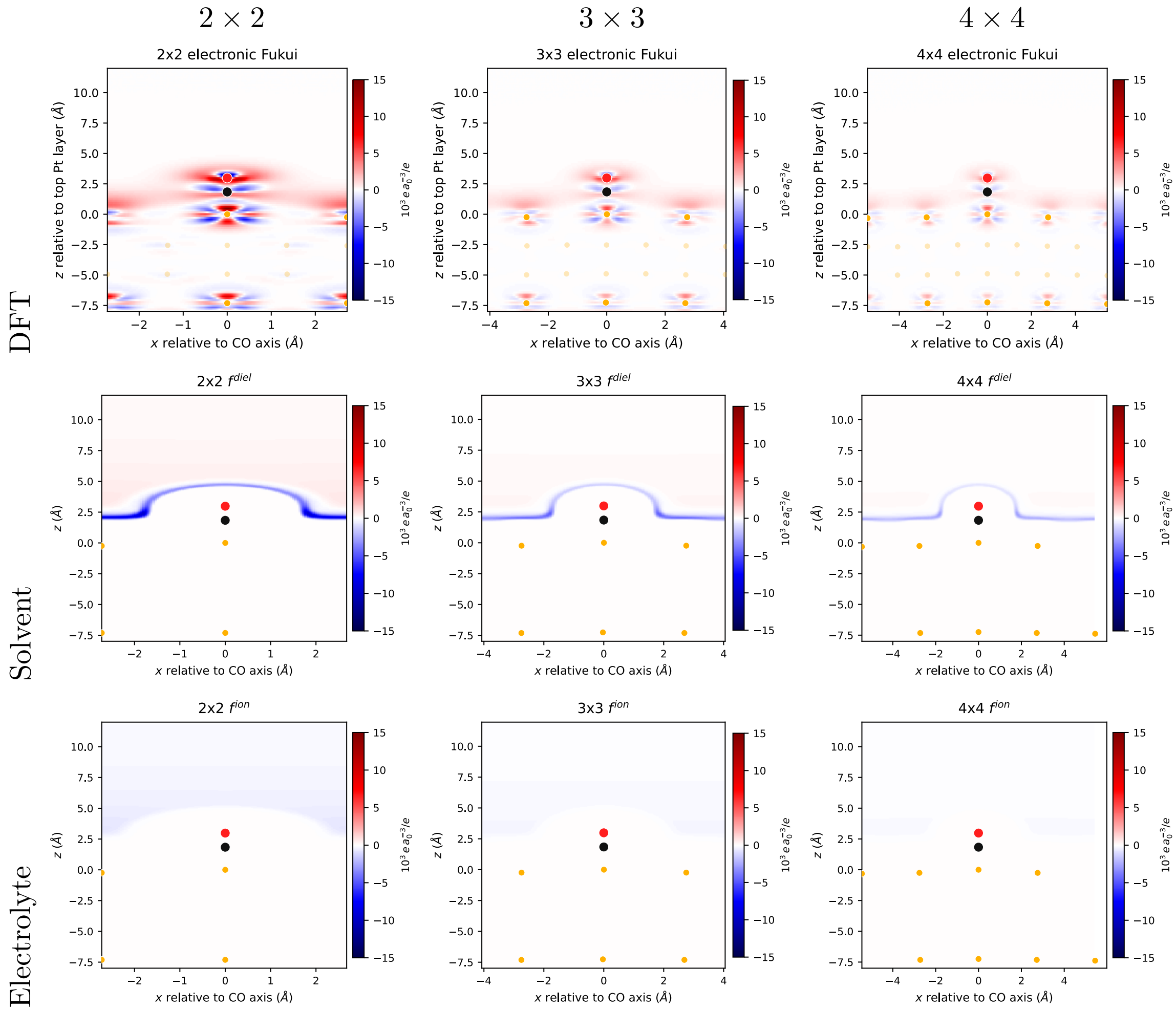

FIG. 7. Fukui-function components for CO/Pt(111) in the 2×2, 3×3, and 4×4 cells. The DFT row shows the electronic Fukui function, $f^{elec}(\mathbf{r}) = \partial\rho^{elec}/\partial N$. The solvent and electrolyte rows show the dielectric and ionic continuum responses, $\partial\rho^{diel}/\partial N$ and $\partial\rho^{ion}/\partial N$, respectively. Black dots mark C atom positions and red dots mark O atom positions. The diffuse-layer Fukui function used in the chemical-hardness analysis is $f^{DL}(\mathbf{r}) = \partial\rho^{diel}/\partial N + \partial\rho^{ion}/\partial N$.

TABLE II. Fixed-coverage control calculations for high-frequency C–O stretch modes. The fixed-coverage 4×4 cell contains four CO molecules, corresponding to 1/4 monolayer coverage. The original 2×2 and one-CO 4×4 results are included as references. $\Delta\omega_{\mathrm{ens}} = \omega^{C} - \omega^{GC}$.

System	Control	ω^{C} (cm^{-1})	ω^{GC} (cm^{-1})	$\Delta\omega_{\mathrm{ens}}$ (cm^{-1})
2×2 original	one CO	2037.539	2018.633	18.906
4×4 original	one CO	2029.679	2025.127	4.552
4×4 fixed coverage	one CO vibrate	2022.620	2019.556	3.064
4×4 fixed coverage	all CO vibrate, highest branch	2039.219	2023.575	15.644

Together, Figs. 6 and 7 show that the states involved in charging are metallic and that the self-consistent electronic and continuum charge responses are localized near the electrified interface. The same local CO perturbation is embedded in progressively larger cells, so the effective vibrating-CO coverage ratio decreases. This spatial picture is consistent with the $1/A$ scaling of the chemical hardness contribution discussed in the main text.

III. FIXED-COVERAGE CONTROL CALCULATIONS

To check whether the decreasing ensemble difference in the one-CO-per-cell series mainly reflects the vibrating-CO coverage ratio or changes in lateral CO-CO interactions, we performed additional diagnostic calculations in a 4×4 cell containing four CO molecules, corresponding to the same 1/4 monolayer total CO coverage as the original 2×2 cell. Two z-direction vibrational subspaces were considered. In the first, all four CO molecules were allowed to vibrate along the surface-normal direction, corresponding to a vibrating-CO coverage ratio of 1/4. This collective calculation is the fixed-coverage analogue of the original 2×2 periodic calculation for the in-phase CO-stretch branch. In the second, only one CO molecule was allowed to vibrate along z, while the other three CO molecules remained fixed, corresponding to a vibrating-CO coverage ratio of 1/16. This local calculation keeps the total CO coverage fixed while reducing the coverage ratio of the vibrating perturbation.

The local single-CO control gives an ensemble shift close to the original one-CO 4×4 result and much smaller than the original 2×2 result. By contrast, the all-CO control

gives a highest-branch shift close to the original 2×2 result. This comparison supports the interpretation that the vibrating-CO coverage ratio, rather than lateral CO-CO coupling, is the dominant factor controlling the ensemble difference for the local stretch.

These fixed-coverage calculations are used only as trend-level controls. They are substantially more expensive than the one-CO calculations and were not converged to the same stringent electronic self-consistency threshold used for the primary finite-difference data. In particular, the multi-CO 4×4 controls were converged to an electronic threshold of approximately 5×10^{-8} rather than 10^{-13}. Because finite differences for vibrational frequencies amplify residual self-consistency noise, the numerical values in Table II should not be interpreted with the same precision as the main frequency data. In the fully converged limit with identical structures and symmetry, the all-CO-mobile in-phase stretch branch in the fixed-coverage 4×4 cell should reproduce the original 2×2 one-CO result. The difference between 15.644 and 18.906 cm^{-1} therefore reflects the numerical precision of this diagnostic control rather than a separate physical effect.

[1] I. Dabo, B. Kozinsky, N. E. Singh-Miller, and N. Marzari, Electrostatics in periodic boundary conditions and real-space corrections, Physical Review B - Condensed Matter and Materials Physics **77**, 1 (2008), arXiv:0709.4647.
